# The Proteasome Inhibitor Bortezomib Induces an Inhibitory Chromatin Environment at a Distal Enhancer of the Estrogen Receptor-α Gene

**DOI:** 10.1371/journal.pone.0081110

**Published:** 2013-12-05

**Authors:** Ginny L. Powers, Prashant Rajbhandari, Natalia M. Solodin, Brant Bickford, Elaine T. Alarid

**Affiliations:** Department of Oncology, McArdle Laboratories for Cancer Research and University of Wisconsin Carbone Comprehensive Cancer Center, University of Wisconsin, Madison, Wisconsin, United States of America; Roswell Park Cancer Institute, United States of America

## Abstract

Expression of the estrogen receptor-α (ERα) gene, *ESR1*, is a clinical biomarker used to predict therapeutic outcome of breast cancer. Hence, there is significant interest in understanding the mechanisms regulating *ESR1* gene expression. Proteasome activity is increased in cancer and we previously showed that proteasome inhibition leads to loss of *ESR1* gene expression in breast cancer cells. Expression of *ESR1* mRNA in breast cancer cells is controlled predominantly through a proximal promoter within ∼400 base pair (bp) of the transcription start site (TSS). Here, we show that loss of *ESR1* gene expression induced by the proteasome inhibitor bortezomib is associated with inactivation of a distal enhancer located 150 kilobases (kb) from the TSS. Chromatin immunoprecipitation assays reveal several bortezomib-induced changes at the distal site including decreased occupancy of three critical transcription factors, GATA3, FOXA1, and AP2γ. Bortezomib treatment also resulted in decreased histone H3 and H4 acetylation and decreased occupancy of histone acetyltransferase, p300. These data suggest a mechanism to explain proteasome inhibitor-induced loss of *ESR1* mRNA expression that highlights the importance of the chromatin environment at the −150 kb distal enhancer in regulation of basal expression of *ESR1* in breast cancer cells.

## Introduction

Expression of ERα in breast cancer is an important clinical determinant of therapeutic strategies. While assessment of ERα protein by immunohistochemistry is the gold standard, quantitative reverse transcriptase PCR assays (qRT-PCR) that incorporate *ESR1* mRNA expression, such as Oncotype Dx and Mammoprint, are gaining utility in predicting response to hormonal and chemotherapies [1]–[3]. Additionally, targeted regulation of *ESR1* mRNA offers an alternative or complementary approach to existing therapies directed at ERα protein and activity [4]. These clinical developments highlight the importance of understanding the control of *ESR1* gene expression in breast cancer cells.

The *ESR1* gene locus is one of the most complex genes in the genome, which makes it challenging to study [5]. It is 450 kb in size and is controlled by seven different promoters, A-E2. Each promoter is regulated in a tissue specific manner, and generates a transcript with a unique 5′-untranslated region. Ultimately, these varying transcripts are spliced to form a single mRNA [5]. The current understanding of *ESR1* gene regulation comes primarily from analysis of promoter usage [6]–[10]. In cell models of ERα-expressing breast tumors, *ESR1* mRNA expression is driven predominantly by the proximal A promoter that encompasses −163/+1 base pairs relative to the transcription start site (TSS) [5]. Conventional reporter gene assays, however, show generally weak activity of this promoter in ERα-expressing breast cancer cells suggesting the involvement of additional elements that are absent in this type of analysis [11], [12].

The 26S proteasome is the primary regulator of ERα protein [13]. Blockade of proteasome activity with various proteasome inhibitors results in an increase in ERα protein in short term experiments [14]–[16]. In contrast, chronic proteasome inhibition (24 hours or more) leads to a near complete loss of ERα [17]. The loss of ERα results from transcriptional repression of the *ESR1* gene as demonstrated by decreases in nascent and steady state levels of *ESR1* mRNA. Indeed, *ESR1* mRNA levels are reduced by as much as 90% in multiple ERα-expressing models (breast, uterine and pituitary) following treatment of cells with bortezomib, a clinical proteasome inhibitor. In the previous study, we noted that although *ESR1* mRNA expression was severely diminished, the level of RNA Polymerase II (RNA PolII) on the proximal promoter was not correspondingly decreased. Moreover, while loss of ERα protein induced by bortezomib would be expected to result in a general inhibition of ERα target gene expression, both gains and losses of gene expression were observed. These data demonstrate that proteasome inhibitors modulate gene expression in breast cancer cells, but how these pharmacologic agents might regulate *ESR1* mRNA remains unclear [17], [18].

Existing models of *ESR1* gene silencing or transcriptional repression identify the *ESR1* proximal promoter as the major regulatory element [19]–[23]. Here, we find that bortezomib treatment selectively targets an *ESR1* distal enhancer (ENH1) located ∼150 kb away from the TSS. Moreover, the results point to a set of bortezomib-induced chromatin modifications consistent with enhancer inactivation at this site. Together, these data support the idea that *ESR1* gene expression in breast cancer cells can be controlled via pharmacological targeting of distal regulatory elements. In addition, they provide evidence that treatment of cells with bortezomib, an established proteasome inhibitor, can alter histone posttranslational modifications to regulate the chromatin environment of an *ESR1* gene enhancer.

## Materials and Methods

### Cell Culture and Drug Treatment

MCF7 cells were maintained as previously described [17]. For all experiments, cells were maintained in phenol-red free DMEM supplemented with 10% charcoal dextran stripped fetal bovine serum [24], 1 mM sodium pyruvate, 1000 U/ml penicillin, and 1000 mg/ml streptomycin (Gibco BRL). Culture conditions were maintained at 10% CO_2_ and 37°C in a water-jacketed incubator (Forma Scientific). Cells were treated with 30 nM bortezomib (gift from Dr. Shigeki Miyamoto) for 24 hours, unless otherwise indicated.

### Western Blot

Western blots were performed as previously described [17], [25]. Cells were lysed directly in 2X sample buffer (62.5 mM Tris-Cl, pH 6.8, 10% glycerol, 2% SDS, 5% β-mercaptoethanol, bromophenol blue) and boiled for 10 minutes. Protein concentration was determined using an RC DC Protein Assay kit (Bio-Rad) as per manufacturer’s instructions. Samples were read on a Genesys 5 spectrophotometer (Spectronic). Proteins (80–100 ug) were electrophoretically transferred using a Trans-blot Cell (Biorad) to nylon membrane (Immobilon-P, Millipore) in a Tris-glycine transfer buffer with 20% methanol. Information on the primary and secondary antibodies is provided in Table S1. Enhanced chemiluminescence (GE Healthcare Bio-Sciences Corp.) was used for protein visualization on X-ray film (Kodak).

### Quantitative Reverse-transcriptase PCR (qRT-PCR)

RNA was isolated with an RNeasy isolation kit (Qiagen) as per the manufacturer’s instructions with the inclusion of an on-column DNase treatment. RNA concentration was measured using a Nanodrop-1000 (Thermo Scientific) and 1 µg was reversed transcribed using iScript cDNA synthesis kit (Bio-Rad). Cycling parameters for reverse transcription were 25°C for 5 minutes, 42°C for 30 minutes, 85°C for 5 minutes and a final hold at 20°C. A myIQ Single Color Real-Time PCR detection system (Bio-Rad) was used for all qRT-PCR. For qRT-PCR of mRNA, the cycling parameters included a 5 minute initial denaturation step at 95°C followed by 40 cycles of denaturation at 95°C for 15 seconds and combined annealing, and elongation steps [25]. A melt curve step was performed to ensure the amplification of a single product. Ribosomal P0 mRNA served as the internal control. Each well contained 1x Sybr Green Master mix (Biorad), 10 ng of cDNA, and 100 nM of the indicated primer pair in final volume of 20 µL. Primer sequences and annealing temperatures are shown in Table S2.

### DNase Sensitivity Assay

DNase sensitivity assays were performed as described previously [26], [27]. MCF7 cells were treated with vehicle or 30 nM bortezomib for 24 hours in estrogen-deprived media. Cell pellets were resuspended in 4X pellet cell volume of lysis buffer (10 mM Tris-Cl, pH 7.5, 10 mM NaCl, 3 mM MgCl_2_, 0.05% NP40) and incubated on ice for 10 minutes. Nuclei were isolated by centrifugation at 1000 rpm for 2 minutes and washed once with digestion buffer (50 mM Tris-Cl, pH 7.5, 100 mM NaCl, 10 mM MgCl_2_, 1 mM DTT). Nuclei were resuspended in digestion buffer and aliquoted into 2 samples (uncut and cut). Based on initial optimization experiments with varying concentrations of DNAse, three Kunitz units of RNAse-free DNase (Qiagen) were added to vehicle and bortezomib-treated samples followed by incubation at 37°C for 5 minutes. The reaction was stopped by addition of 15 mAU Proteinase K (Sigma) and incubation at 65°C for 15 minutes. DNA was purified from samples using a DNAeasy kit (Qiagen) following manufacturer’s protocol. Quantitative real-time PCR was carried using 20 ng of DNA with the primers shown in Table S3. PCR conditions were identical to those used in ChIP assays and are described below. Relative DNase sensitivity was calculated for three independent experiments as DNase sensitivity = 2^((Ct cut–Ct uncut))^.

### Chromatin Immunoprecipitation (ChIP)

ChIP was performed as described in previous studies [17]. Two 10-cm plates were used for each treatment group. Twenty four hours after treatment, media was aspirated, rinsed with PBS, and crosslinked with 1.5% formaldehyde for 15 minutes at 37°C. Cells were harvested and pelleted by centrifugation at 3000 rpm for 5 minutes at 4°C. Following two washes with ice-cold PBS, cells were pelleted and either frozen at −80°C or resuspended in 300 µL of nuclei lysis buffer (50 mM Tris-Cl, pH 8.1, 10 mM EDTA, 1% SDS, 10 µg/mL leupetin (Roche), 10 µg/mL aprotinin, 0.2 mM sodium orthovanadate (Calbiochem), and 2 mM PMSF). After a 10 minute incubation on ice, the cell suspension was sonicated three times on setting 3 at 4°C for 15 seconds with a 550 Sonic Dismembrator (Fisher Scientific) to obtain chromatin fragments in the 500–1000 bp range. Lysate was spun down for 10 minutes at 13,000 rpm at 4°C and 30 µL was frozen at −80°C as the 10% input control. The remainder of the sample was divided into tubes for immunoprecipitation with the indicated antibody and diluted 1∶10 with IP buffer (1% triton-X, 2 mM EDTA, 150 mM NaCl, and 20 mM Tris-Cl, pH 8.0). Lysates were precleared with 2 µg of herring sperm DNA, 5 µg of BSA and 20 µL of 50% slurry of protein A sepharose (GE Healthcare Bio-Sciences Corp.) or protein A/G agarose (Santa Cruz) beads depending on the antibody. Immunoprecipitations were carried out overnight at 4°C. The specific conditions for each antibody including concentration and amount of lysate used are shown in Table S4. Beads were harvested by centrifugation for 5 minutes at 5000 rpm at 4°C, washed for 10 minutes rotating at 4°C with 1 mL of wash buffer I, and then spun at 5000 rpm for 5 minutes at 4°C. The washes were repeated as follows: Wash buffer II-A for ChIP using p300 antibody and Wash buffer II-B for all other antibodies, Wash buffer III and twice with TE wash buffer. Buffer compositions are as follows:

Wash buffer I: 20 mM Tris Cl pH 8, 2 mM EDTA, 150 mM NaCl, 1% triton-X, 0.1% SDS

Wash buffer II-A: 20 mM Tris Cl pH 8, 2 mM EDTA, 500 mM NaCL, 1% triton-X

Wash buffer II-B: 20 mM Tris Cl pH 8, 2 mM EDTA, 500 mM NaCL, 1% triton-X, 0.1% SDS

Wash buffer III: 10 mM Tris Cl pH 8, 1 mM EDTA, 0.25 M LiCl, 1% NP-40, 1% dioxycholate

TE wash buffer: 10 mM Tris Cl pH 8, 1 mM EDTA

After a final TE wash, the complexes were extracted from the beads with a 30 minute incubation and two additional 10 minute incubations at room temperature with 75 µL of 1% SDS and 0.1 M NaHCO3. Samples were spun down at 5000 rpm for 5 minutes, and supernatants were collected and pooled. Extracted and 10% input control samples were covered with mineral oil and heated overnight at 65°C to reverse DNA: protein crosslinks. DNA was purified with a PCR Purification Kit (Qiagen), eluted in 50 µL of elution buffer, and then frozen at −20°C or used immediately for quantitative PCR (qPCR). Reactions for quantitative real-time PCR contained 1x IQ Sybr Green Supermix (Bio-Rad), 200 nM of primers and 1 µL input or 2–4 µL of IP. DNA levels were measured using the myIQ Real-Time PCR detection system (Bio-Rad) using a program that consisted of a single cycle of 95°C for 3 minutes, followed by 40 cycles of 95°C for 15 s, and 55–60°C, depending on the antibody, for 1 minute. Primer sequences and annealing temperatures are listed in Table S3. A final denaturation step of 95°C for 1 minute was followed by a melt curve ranging from 55–95°C increasing 0.5°C per cycle for 30 seconds each. Data were analyzed based on the percent input: (100* 2^∧^(Ct_input_–Ct_IP_))/z where z = IP [(µL loaded qPCR/µL eluted during DNA purification)*(µL in IP/total lysate)]/input [(µL loaded qPCR/µL eluted during DNA purification)*(µL in input/total lysate)].

### Statistical Analysis

Experimental results reflect the analysis of a minimum of three independent experiments. Student’s paired t-tests, ANOVA, and Tukey’s tests were performed using Graphpad Prismn (GraphPad Software, Inc. La Jolla, CA). Wilcoxon Signed Rank test was performed using MStat [28]. Statistical tests are indicated in each figure legend.

## Results

### Bortezomib Treatment Reversibly Decreases ESR1 mRNA Expression

**Figure 1:**
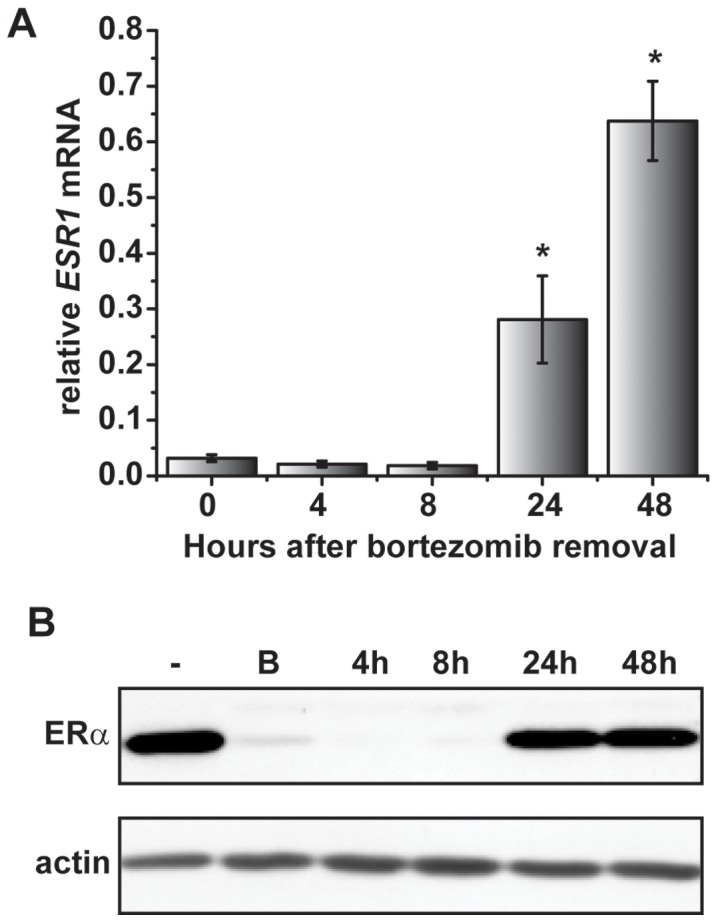
The effects of proteasome inhibition on ERα expression are reversible. *A*) MCF7 cells were treated with bortezomib for 24 hours and harvested immediately (t = 0) or at the indicated time from 4 to 48 hours post bortezomib removal. *ESR1* mRNA levels were measured by qRT-PCR. Data are shown relative to an untreated control before bortezomib-treatment, which was set at 1. Results are representative of three independent experiments and are shown as mean ± SEM. For statistical analysis, ANOVA was performed using the ΔCt values followed by a post hoc Tukey’s test to compare each point to the 0 hour. Statistically significant values (p<0.05) are indicated with an *. *B*) Western blots were performed on whole cell lysates from cells that were treated with vehicle (−) or bortezomib (B) for 24 hours (24 h). After 24 hours, bortezomib was removed by washing cells twice with PBS and replacing media. Cells were then harvested at the indicated times between 4 and 48 hours after bortezomib removal. Blots were probed with antibodies for ERα, with actin serving as a loading control.

Chronic proteasome inhibition leads to significant loss in *ESR1* mRNA expression after 24 hours [17]. To explore the underlying mechanism, experiments were initially performed to ask whether the effect of bortezomib on *ESR1* mRNA was permanent or transient. MCF7 cells were chosen as the preferred ERα-expressing cell model since we previously showed that bortezomib represses *ESR1* mRNA expression [17] and characterized 5′ regulatory elements governing *ESR1* mRNA in this cell line [29]. Cells were treated for 24 hours with bortezomib followed by washing and media replacement. *ESR1* mRNA expression was assessed at various times subsequent to media change and evaluated relative to levels in control samples that were not treated with bortezomib (Fig. 1). Experiments were performed in the absence of estrogen since estrogen can independently repress *ESR1* mRNA [17], [29]. As expected [17], treatment with bortezomib led to an approximate 95% decrease in *ESR1* mRNA expression relative to controls (Fig. 1*A*). Following removal of bortezomib, *ESR1* mRNA expression partially recovered at 24 hours and continued to increase at 48 hours (Fig. 1*A*). Recovery of *ESR1* mRNA expression was reflected by coordinate increases in ERα protein as shown by Western blot analysis (Fig. 1*B*). These data show that the effect of bortezomib on *ESR1* mRNA is reversible, indicative of a non-stable mechanism governing basal *ESR1* transcription.

### A Repressive Chromatin Environment is Established on the ESR1 Enhancer Region with Bortezomib Treatment

**Figure 2:**
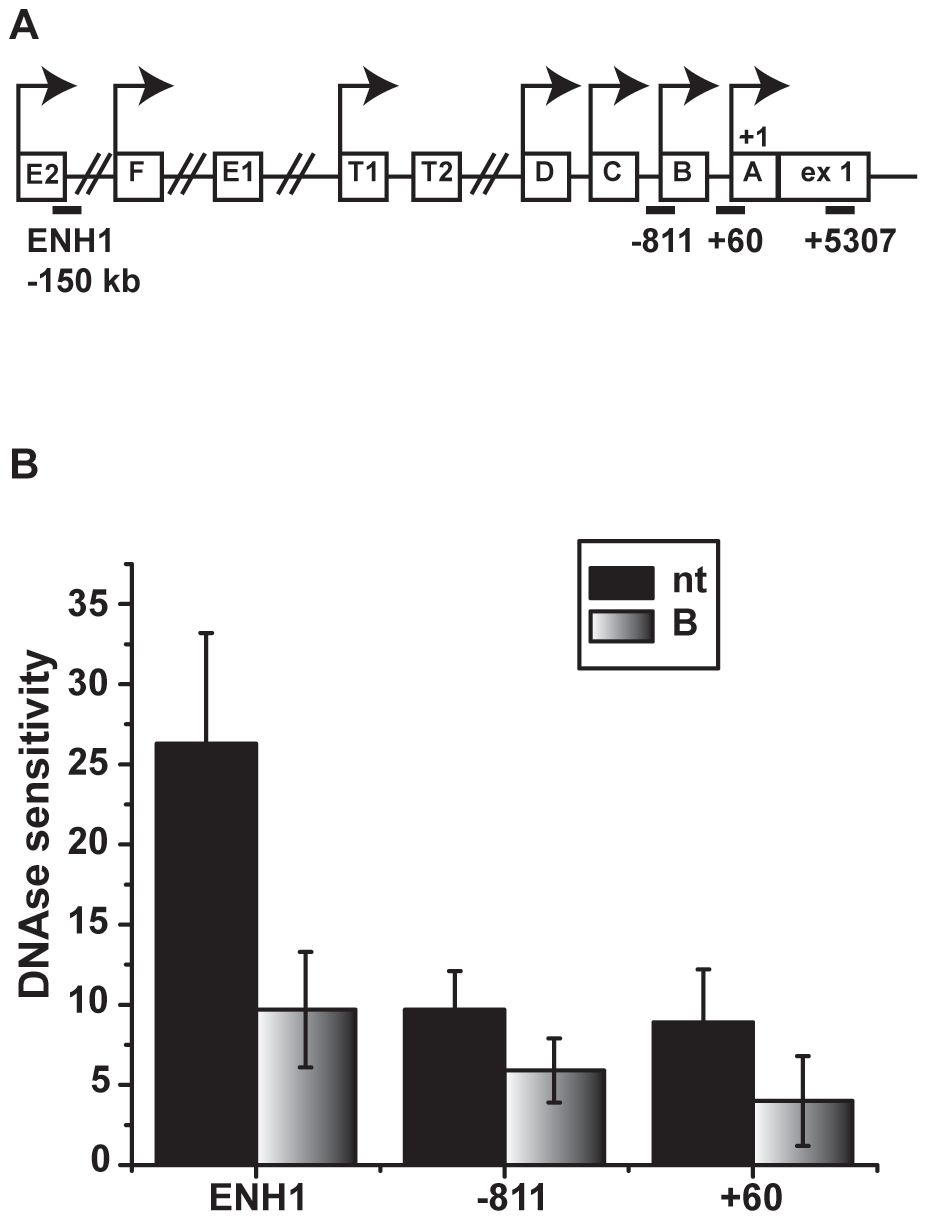
DNase sensitivity is reduced in the *ESR1* enhancer region with proteasome inhibition. *A*) Schematic of *ESR1* promoter structure depicting the location of primers used for detecting DNase sensitivity and ChIP analysis *B)* Nuclei were isolated from MCF7 cells treated for 24 hours with vehicle or 30 nM bortezomib. Isolated nuclei were either left undigested or digested with DNase for 5 minutes, followed by incubation with Proteinase K to stop the DNase reaction, as described in Materials and Methods. DNA was purified and q-PCR was performed with primers to the indicated regions. Data are shown as the 2^∧(Ct cut–Ct uncut)^ and presented as mean ± SEM. Statistical analysis was performed using a Student’s t-test. No statistically significant differences were observed (p>0.05).

The chromatin environment was next examined with focus on the proximal promoter and a distal enhancer (ENH1) located −150 kb from the TSS. The proximal promoter is a major regulatory region governing *ESR1* mRNA in breast cancer [9], [30], [31], and the distal enhancer is involved in regulation of *ESR1* mRNA by estrogen [32]. A schematic of the *ESR1* 5′ regulatory region is shown in Fig. 2*A*. To examine general changes in chromatin at the enhancer and the promoter regions following bortezomib treatment, DNase sensitivity assays were performed. Nuclei isolated from control and bortezomib-treated cells were isolated and exposed to DNAse I. After column purification, q-PCR was performed to quantify protected fragments at the distal region (ENH1; −150 kb) and promoter (+60), as well as a non-specific intervening region (−811) [29]. Bortezomib treatment resulted in an apparent ∼2.5 fold decrease in DNase cleavage at the distal enhancer. While not statistically significant, the trend implied that this region may be protected in bortezomib-treated cells (Fig. 2*B*). In contrast, bortezomib treatment had little impact on the proximal promoter and the intervening region (−811).

**Figure 3:**
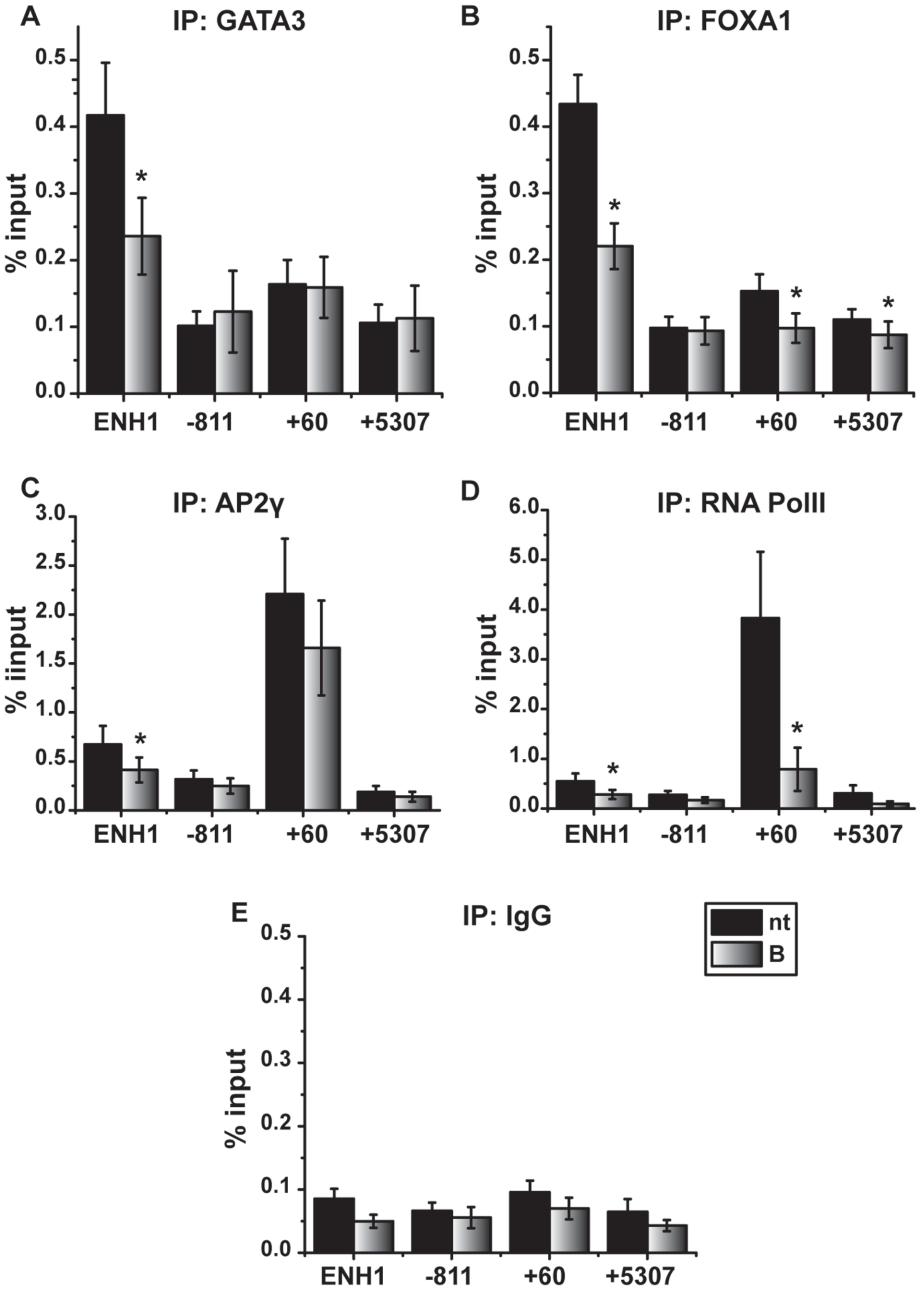
Proteasome inhibition decreases transcription factor occupancy on the *ESR1* enhancer and promoter. MCF7 cells were treated with vehicle (nt) or 30 nM bortezomib (B) for 24 hours and ChIP analyses were performed to examine occupancy at regions of *ESR1* depicted in the diagram in Fig. 2A. Data representing a minimum of three independent experiments are presented as percent of input sample for *A*) GATA3, *B*) FOXA1, *C*) AP2γ, *D*) RNA PolII, and *E*) IgG (control) on the indicated regions. Data are shown as mean ± SEM. Statistical analysis comparing untreated and bortezomib-treated groups were performed using a Student’s paired t-test. Statistically significant differences of p<0.05 are indicated with an asterisk (*).

These data prompted us to ask whether bortezomib could alter other indicators of enhancer activity. Three key transcriptional regulators regulate *ESR1* expression in ER-expressing breast cells *in vitro* and *in vivo*; GATA3, FOXA1, and AP2γ [32]–[34]. Previous studies performed in the presence of estrogen identified FOXA1 and AP2γ occupancy on the *ESR1* proximal promoter and GATA3 binding at the distal ENH1 region [32], [33], [35]–[37]. To evaluate transcription factor (TF) occupancy in the absence of estrogen, chromatin immunoprecipitation (ChIP) assays were performed to assess occupancy of GATA3, FOXA1, and AP2γ in estrogen-deprived medium [32]. Consistent with previous reports, GATA3 occupancy was greatest at the distal enhancer (ENH1) (Fig. 3*A*) [32], and occupancy of AP2γ was greatest at the proximal promoter (+60) (Fig. 3*C*) [35]. Like GATA3, FOXA1 occupancy was highest at the distal ENH1 site (Fig. 3*B*). RNA PolII binding was highest at the proximal promoter (Fig. 3*D*), consistent with previous studies [29]. Non-specific IgG (Fig. 3*E* *)* and two non-regulated sites (−811 and +5307) served as controls [29].

When cells were treated with bortezomib, significant changes in TF and RNA PolII occupancy were observed on the distal enhancer. GATA3 and FOXA1 binding to ENH1 decreased by approximately two-fold relative to untreated controls. AP2γ occupancy also declined, but to a lesser extent. A significant decrease in FOXA1 occupancy was also observed on the proximal promoter (+60) and the coding region (+5307), though it should be noted that the level of occupancy of FOXA1 at these regions was generally low and similar to the level observed at the intervening region at −811 and non-specific IgG controls. Bortezomib also induced an approximate 4-fold decrease in RNA PolII binding at the promoter as well as the ENH1 region relative to controls (Fig. 3*D* *,* [17]).

Examination of GATA3, FOXA1, and AP2γ mRNA and protein indicated that the decreased occupancy was unlikely due to changes in TF expression (Fig. S1). FOXA1 and AP2γ levels were unchanged by bortezomib treatment. Although GATA3 protein declined in the presence of bortezomib, stable re-introduction of GATA3 was unable to rescue *ESR1* mRNA expression (data not shown). In all, these data indicate that bortezomib diminishes TF occupancy at both the proximal and distal region, but the magnitude of changes were greatest at the ENH1 region.

### Proteasome Inhibition Decreases p300 Occupancy and Histone Acetylation on the ESR1 ENH1

**Figure 4:**
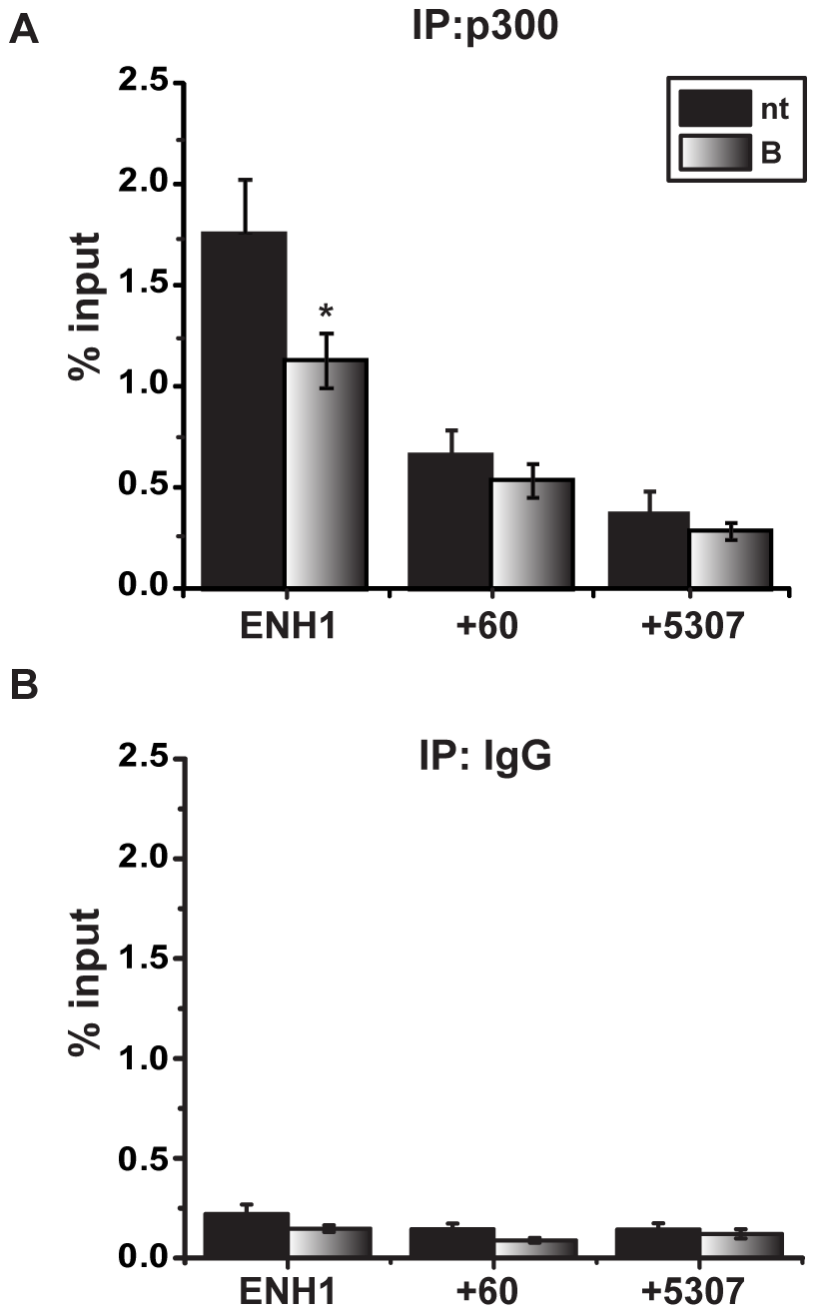
Proteasome inhibition decreases p300 occupancy on the *ESR1* enhancer. MCF7 cells treated with vehicle (nt) or 30 nM bortezomib (B) for 24 hours and ChIP assays were performed to assess occupancy of p300 at the indicated region. Immunoprecipitation was performed using antibodies for *A*) p300 and *B*) IgG, as a control. Data are presented as percentage of the input sample. The data represent the average of a six independent experiments ± SEM. Statistical analysis was performed to compare untreated and bortezomib-treated samples using a Student’s paired t-test. Statistically significant differences of p<.05 are indicated with an asterisk (*).

**Figure 5:**
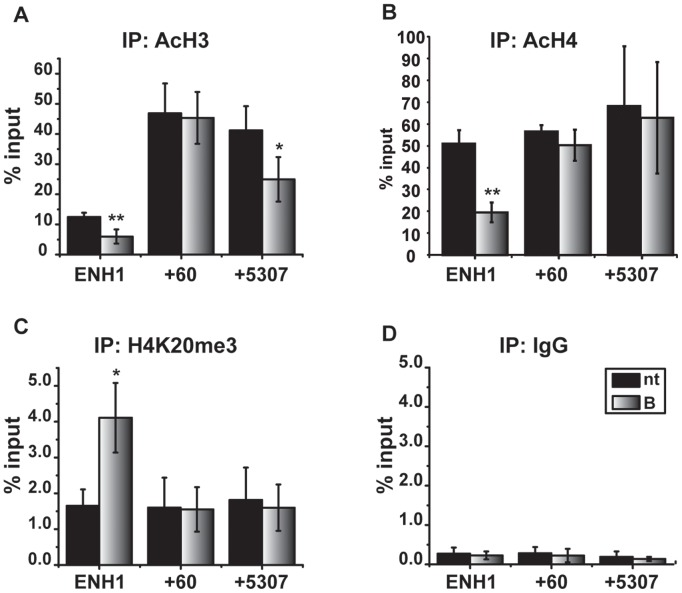
Proteasome inhibition decreases histone acetylation and increases H4K20me3 on the distal *ESR1* enhancer. MCF7 cells were treated for 24 hours with vehicle (nt) or 30 nM bortezomib (B), and ChIP was performed as described in methods using antibodies to *A*) pan acetylated histone 3 (AcH3), *B*) pan acetylated histone 4 (AcH4), *C*) histone 4 lysine 20 tri methylation (H4K20me3), and *D*) IgG as control. Data are presented as percentage of input sample and represent a minimum of three independent experiments. Error bars indicate the SEM, and an asterisk (*) indicates a statistically significant difference between vehicle and bortezomib-treated samples using a Student’s paired t-test. * p<0.05, ** p<.01.

The histone acetyltransferase, p300, also marks active enhancers [38], [39] and has been shown to interact with GATA3 in other contexts [32], [40], [41]. Under control conditions, p300 occupancy is greatest on the ENH1 region. After 24 hours of bortezomib treatment, p300 occupancy on ENH1 significantly decreased with no changes at the promoter (+60), or the coding region (+5307) (Fig. 4*A*). An IgG control was unchanged with treatment (Fig. 4*B*). Consistent with the loss of p300, acetylation status of histone H3 (AcH3) and histone H4 (AcH4) was also decreased at the ENH1 region (Fig. 5*A–B*). In addition, AcH3 also significantly decreased in the coding region (+5307). Control ChIP analyses at the same time point showed that levels of total histones H3 and H4 were unchanged in control and treated groups; thus, decreases in histones H3 or H4 cannot account for decreases in acetylation (Fig. S2 *A*–*B*). Similarly, IgG controls were also unchanged by treatment (Fig. 5*D*). It is notable that AcH3 and AcH4 on the promoter were relatively high in both the presence and absence of bortezomib, despite inhibition of *ESR1* mRNA expression.

ChIP analyses were extended to include additional repressive chromatin modifications including histone H3 lysine 9 trimethylation (H3K9me3), histone H3 lysine 27 trimethylation (H3K27me3), and histone H4 lysine 20 trimethylation (H4K20me3). H3K9me3 and H3K27me3 are typically associated with gene silencing [42], while H4K20me3 has been linked with decreased, but not silenced, gene expression [43]. Bortezomib significantly increased H4K20me3 in the enhancer region (Fig. 5*C* *),* while silencing marks, H3K9me3 and H3K27me3 [42], were not altered on any of the *ESR1* regions tested (Fig. S2 *C*–*D*). These results are consistent with the establishment of a repressive, but not silenced chromatin environment on the distal enhancer of the *ESR1* gene.

## Discussion

**Figure 6:**
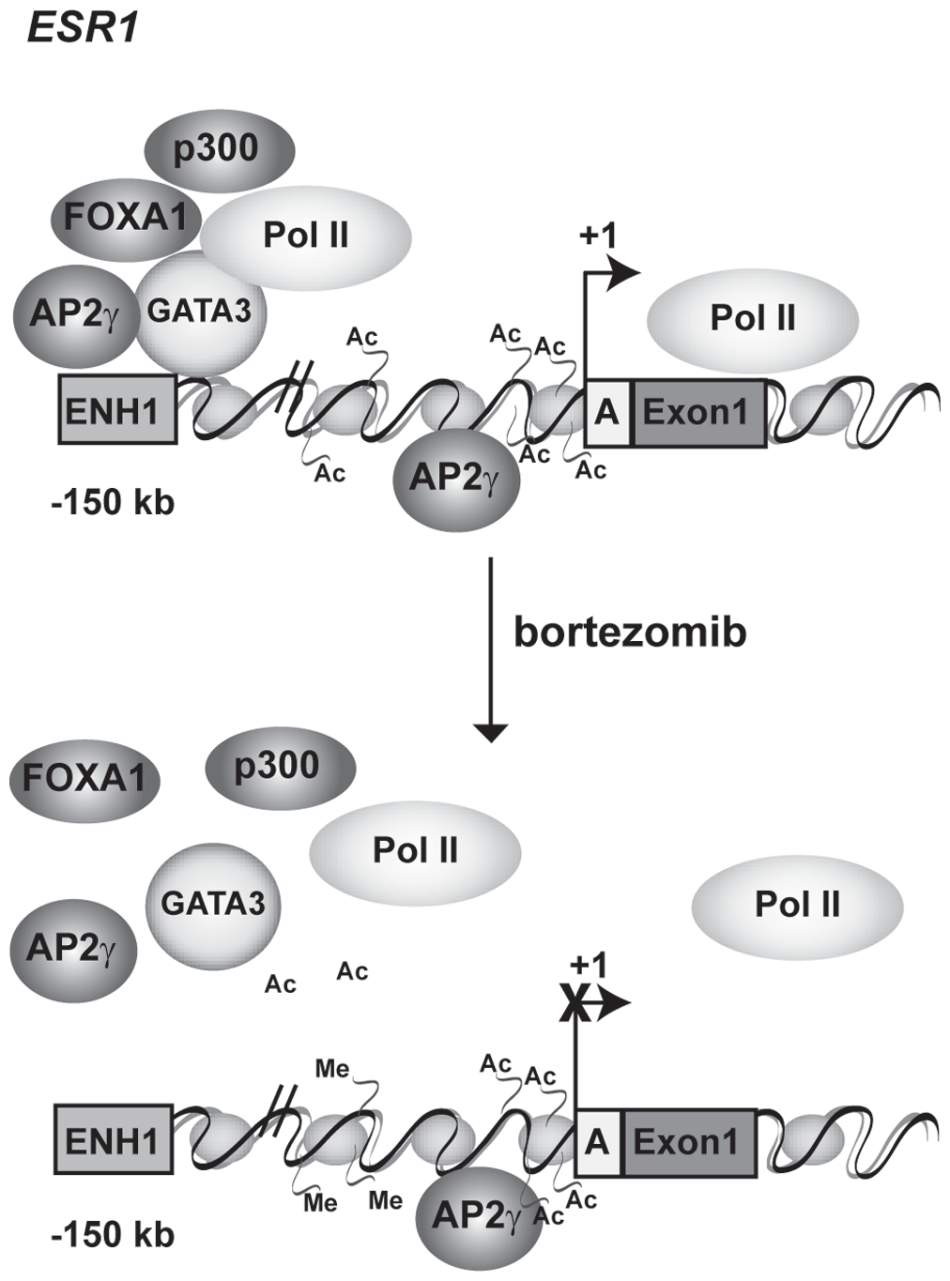
Model of *ESR1* regulation by proteasome inhibitors. Under basal conditions in the absence of estrogen, the *ESR1* distal enhancer is acetylated on histones H3 and H4, and is occupied by FOXA1, AP2γ, GATA3, p300 and RNA PolII. Histone H3 and H4 are also acetylated on the proximal promoter which is occupied by RNA PolII and AP2γ. After the addition of bortezomib, the distal enhancer exhibits decreased histone acetylation and increased histone methylation. Occupancy of FOXA1, AP2γ, GATA3, p300 and RNA PolII decreases on the distal enhancer. In contrast, histone acetylation, methylation, and AP2γ occupancy on the proximal promoter are unchanged, but RNA PolII occupancy decreases. These data support a model where bortezomib-induced changes in the chromatin environment around the distal enhancer regulate *ESR1* expression in ER+ breast cancer cells.

Despite the importance of ERα in breast cancer diagnostics and therapy, our understanding of regulation of the *ESR1* gene in ER+ cancer cells and by cancer therapeutics is limited due to its complex gene organization. In this study, we examined effects of the proteasome inhibitor bortezomib on two known regulatory regions of the *ESR1* gene; the proximal promoter and the distal enhancer (ENH1). The data show that bortezomib induced a set of changes to the chromatin environment, which predominantly impacted the distal enhancer region. These changes include decreases in occupancy of TFs and p300, as well as decreases in histone acetylation and increases in histone methylation. Together, these bortezomib-induced changes are consistent with inactivation of the distal enhancer. Based on these data, we propose the following model (Fig. 6). In the absence of estrogen and bortezomib, *ESR1* mRNA is expressed and the proximal promoter is active and occupied by RNA PolII. Histones H3 and H4 are acetylated and AP2γ is bound at the promoter. At the distal ENH1 enhancer, GATA3, FOXA1, and p300 are bound in addition to AP2γ and RNA PolII. Histones H3 and H4 are acetylated, although AcH3 and AcH4 levels are lower at ENH1 than the promoter region. Upon Bortezomib treatment, TFs and RNA PolII are ejected, H4K20me3 increases and AcH3 and AcH4 decreases at ENH1. In contrast, changes occurring at the promoter region were limited to decreases in RNA PolII and FOXA1. These bortezomib-induced changes and the resultant inhibitory chromatin environment at the distal enhancer could account for the loss of *ESR1* mRNA expression. These studies expand the functional importance of a distal enhancer as a target for pharmacologic manipulation, and highlight the potential role of chromatin modification in this region in the basal expression of *ESR1* mRNA in ER-positive breast cancer cells.

Our results reinforce the role of both the distal ENH1 enhancer and proximal promoter in *ESR1* mRNA expression in MCF7 cells. Like the proximal promoter, the distal enhancer is occupied by several transcription factors, including GATA3, FOXA1, and histone acetyltransferase, p300. AP2γ occupies both the distal enhancer and the proximal promoter although its highest level of occupancy is on the promoter. To our knowledge, these studies are the first to show that FOXA1 and AP2γ occupy regions outside the A promoter with the important distinction being that the present study was performed in the absence of estrogen [33], [35]. Proteasome inhibition resulted in decreased occupancy of all three factors at ENH1. Moreover, the loss of transcription factor binding coincided with diminished p300, AcH3 and AcH4 and increased H4K20me3, which is consistent with a general repressive chromatin environment in this region. Interestingly, the proximal promoter remained in an active configuration with relatively high levels of AcH3 and AcH4. These data suggest that despite a transcriptionally-permissive status at the promoter, *ESR1* mRNA expression is more closely correlated with the chromatin modifications at the distal site. Thus, *ESR1* expression may depend on an open chromatin environment at the distal enhancer in addition to the promoter, and therapies that convert the distal region to a closed state can significantly impact ERα status in breast cancer cells.

Only a few studies have explored distal sites that regulate *ESR1* mRNA expression. The Brown group identified an enhancer region, “E0” that is approximately 3800 bp upstream of the TSS near the D promoter, using traditional reporter assays with fragments of the *ESR1* 5′ regulatory region linked to luciferase [44]. Subsequently, based on ChIP data, Eeckhoute et al. identified GATA3 binding to the distal ENH1 enhancer [32]. Previous work from our laboratory also identified activators, p300 and AIB1, binding to the same enhancer upon estrogen treatment as a component of a repressive mechanism [29]. The studies presented here provide additional evidence for a functional role of the chromatin environment of the distal enhancer in controlling high levels of expression of *ESR1* mRNA in ER-expressing breast cancer cells. We observed that repression of this region corresponds with a loss of several chromatin marks associated with active enhancers. Studies by Davidson’s group showed that treatment of ER-negative cells with HDAC inhibitors and 5-azacytosine, can relieve transcriptional silencing of *ESR1* and cause expression of ERα [23], [45]. The authors attributed the re-expression of *ESR1* mRNA to occupancy of factors on the proximal promoter. The data shown here suggest that these chromatin-targeting agents may likewise affect the distal enhancer. Indeed, work from our lab indicates that the distal and proximal promoter co-regulate *ESR1*expression [29]. For example, estrogen-induced repression of *ESR1* mRNA involves recruitment of factors to both the proximal and the distal regions but chromatin modifications occur primarily at the proximal promoter. Proteasome inhibition likewise induces changes at both sites, but preferentially impacts the distal enhancer where more global changes in TF occupancy and chromatin modifications occur.

The role of GATA3 and FOXA1 in the regulation of *ESR1* mRNA and ERα-mediated transcription is well documented. GATA3 and FOXA1 are correlated with ERα positive breast tumors and are critical in normal mammary gland development in rodent models [46], [47]. The loss of GATA3 expression decreases luminal progenitor cells and also regulates the expression of FOXA1, which suggests that both factors may be important in mammary differentiation [48], [49]. Moreover, both GATA3 and FOXA1 are necessary, in addition to ERα expression, to recover estrogen-responsiveness in breast cells [40]. We noted that bortezomib depleted GATA3 protein, which is consistent with evidence suggesting an important role for GATA3 in *ESR1* mRNA expression in breast cells. However, knockdown of GATA3 did not alter *ESR1* mRNA expression and re-expression of GATA3 did not rescue *ESR1* mRNA expression in the presence of bortezomib in our model (data not shown). This is in contrast to studies by Eeckhoute et al. which showed that GATA3 knockdown resulted in loss of *ESR1* mRNA expression in T47D cells [32]. A possible explanation for the discrepancy could be due to differences in estrogen conditions in the experimental designs. Studies investigating the links between ERα, GATA3, and FOXA1 demonstrate that these factors are involved in complex cross-regulatory loops [32], [33]. GATA3 regulates the expression of both *ESR1* and *FOXA1* mRNA, while ERα regulates *GATA3* mRNA. FOXA1 regulates *ESR1* mRNA but not *GATA3* mRNA. Since estrogen activation of ERα is necessary to engage these regulatory loops, the presence or absence of estrogen can impact the data. Our studies were done in the absence of estrogen since our earlier work indicated that bortezomib and estrogen induce transcriptional repression of *ESR1* mRNA by independent mechanisms. Alternatively, the dependence on GATA3 may be cell-type specific. Proteasome inhibition by bortezomib and another proteasome inhibitor, MG132, causes loss of *ESR1* mRNA in multiple ER-expressing cell lines, including MCF7, T47D, BT474, and PR-1. Thus, it is unlikely that the effects of proteasome inhibition result from activities of individual factors in specific cells. Our data instead support a more generalized mechanism that broadly influences enhancer activity through a combinatorial effect on the chromatin environment.

In summary, this study describes a new mode of chromatin regulation by the proteasome inhibitor bortezomib revealed through analysis of transcriptional repression of *ESR1* mRNA. We find that proteasome inhibition resulted in the loss of active marks surrounding a distal enhancer. These studies highlight the notion that basal regulation of *ESR1* gene expression depends on the chromatin environment and activity of a distal enhancer, which can control expression independent of promoter status. Future targeting of ERα in breast cancer through the controlled expression of *ESR1* mRNA will therefore be improved by broadening our understanding to include distal sites of regulation in addition to promoter analyses.

## Supporting Information

Figure S1 Bortezomib decreases GATA3 but not FOXA1 or AP2γ expression. ***A***) MCF7 cells were treated with vehicle (−) or 30 nM bortezomib (B) for 24 hours and RNA was isolated. Quantitative RT-PCR was run to determine mRNA levels of *GATA3*, *FOXA1*, and AP2γ. Bortezomib-treated samples are presented as fold change relative to control, vehicle-treated samples. Data represent a minimum of three independent experiments and is shown as the mean ± SEM. Statistically significant differences were determined using a Wilcoxon signed rank test. p<0.05 is indicated by *. *B*) Western blots were performed on whole cell lysates treated with bortezomib as in *A*. Blots were probed with antibodies against GATA3, FOXA1, or AP2γ. Blots were stripped and reprobed with actin as a loading control. Data shown are representative results from a minimum of three independent experiments.
(TIF)

Figure S2 Proteasome inhibition does not alter total H3 or H4 or tri-methylation of H3K9 or H3K27. MCF7 cells were treated for 24 hours with vehicle (nt) or 30 nM bortezomib (B), and ChIP assays were performed using antibodies for *A*) total histone 3 (H3), *B*) total histone 4 (H4), and *C*) H3K27me3, *D*) H3K9me3. IgG controls are shown in Fig. 5. Data are presented as percent input and represent a minimum of three independent experiments. No statistically significant differences were found (p>0.05).
(TIF)

Table S1 Antibodies used for Western Blots. Primary antibodies to the indicated proteins of interest are listed with the specific clone in parenthesis. The Catalog number given is specific for the Company from which the antibody was purchased. The Concentration indicates the dilution of primary antibody in a solution of 5% milk that was used in the Western blot analysis.
(DOCX)

Table S2 Primers used for Quantitative Reverse Transcriptase PCR (qRT-PCR) of mRNA. Listed are the accession number, location and sequences of primers used for qRT-PCR. Temp. indicates the optimized annealing temperature used for each primer set.
(DOCX)

Table S3 Primers used for ChIP and DNAse sensitivity assays. Primers used in quantitative PCR analyses of ChIP and DNAse sensitivity assays for the *ESR1* gene are shown. The name of the primer indicates its location based on base pair distance 5′ of the TSS. Temp. indicates the optimized annealing temperature for each primer pair.
(DOCX)

Table S4 Antibody conditions for ChIP. Shown is a list of antibodies with corresponding catalog number and commercial vendor. Also included is the amount of antibody used for the indicated volume of lysate out of a total of 300 µl of total cell lysate.
(DOCX)
