# Supplementary figures and images for: The Proteasome Inhibitor Bortezomib Induces an Inhibitory Chromatin Environment at a Distal Enhancer of the Estrogen Receptor-α Gene
Source: PLoS One. 2013 Dec 5;8(12):e81110. doi: 10.1371/journal.pone.0081110 (PMC3855213; doi:10.1371/journal.pone.0081110)

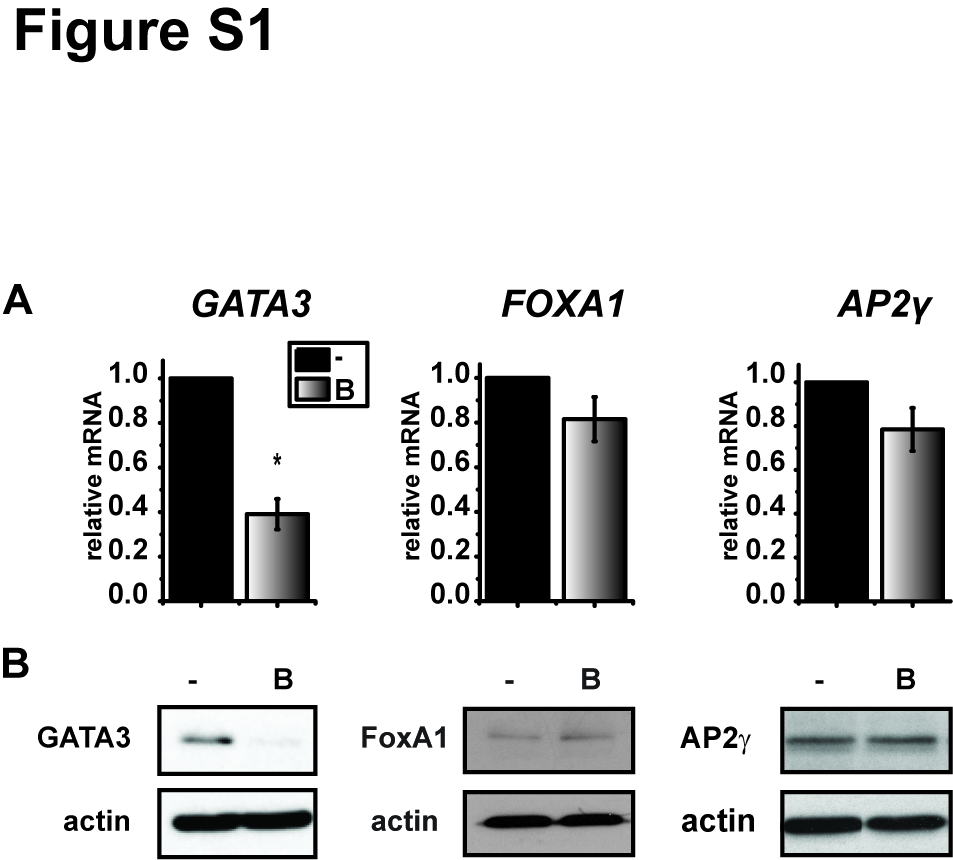

Supplement: Figure S1 — Bortezomib decreases GATA3 but not FOXA1 or AP2γ expression. A) MCF7 cells were treated with vehicle (−) or 30 nM bortezomib (B) for 24 hours and RNA was isolated. Quantitative RT-PCR was run to determine mRNA levels of GATA3, FOXA1, and AP2γ. Bortezomib-treated samples are presented as fold change relative to control, vehicle-treated samples. Data represent a minimum of three independent experiments and is shown as the mean ± SEM. Statistically significant differences were determined using a Wilcoxon signed rank test. p<0.05 is indicated by *. B) Western blots were performed on whole cell lysates treated with bortezomib as in A. Blots were probed with antibodies against GATA3, FOXA1, or AP2γ. Blots were stripped and reprobed with actin as a loading control. Data shown are representative results from a minimum of three independent experiments. (TIF) [file pone.0081110.s001.tif]

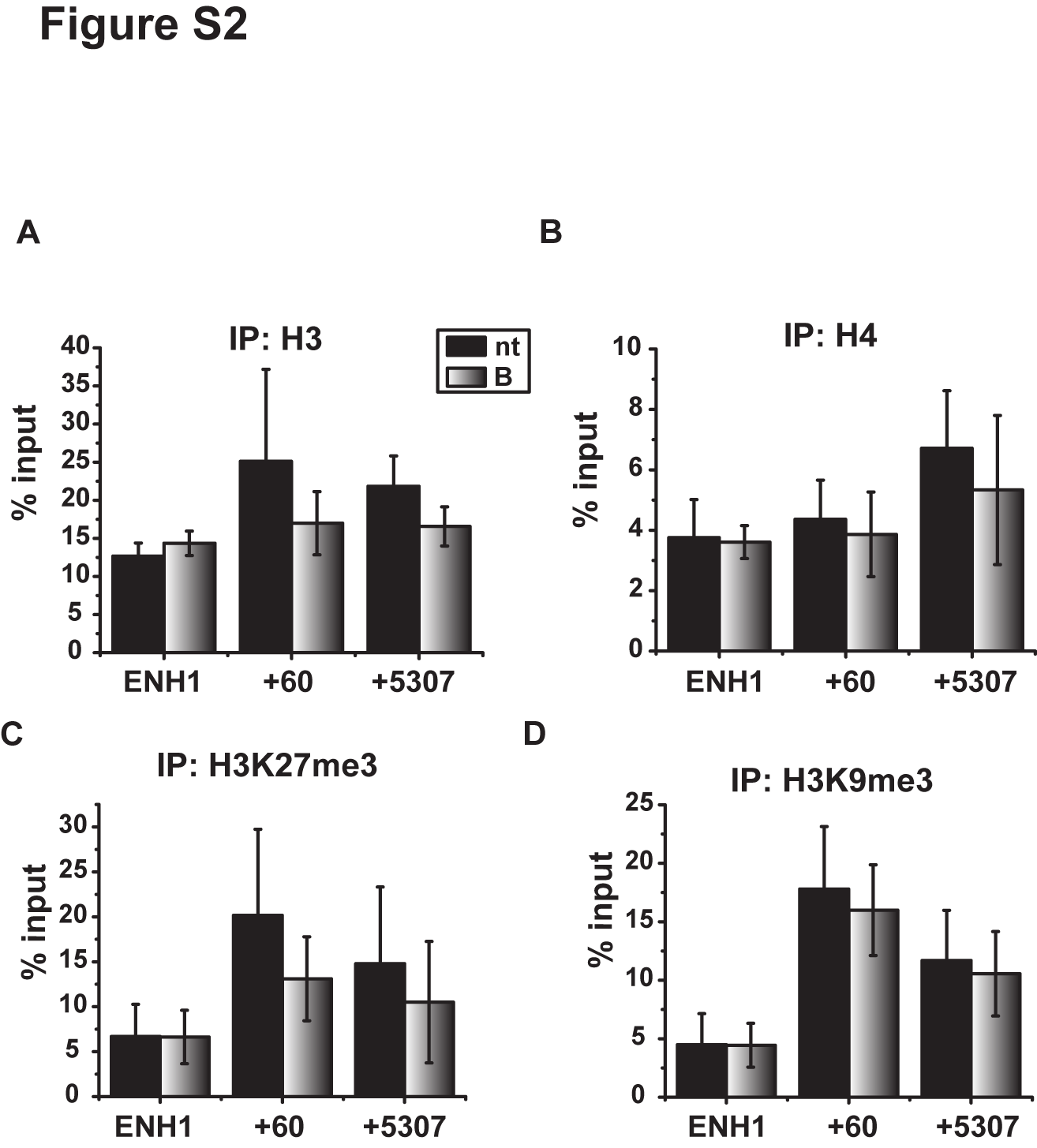

Supplement: Figure S2 — Proteasome inhibition does not alter total H3 or H4 or tri-methylation of H3K9 or H3K27. MCF7 cells were treated for 24 hours with vehicle (nt) or 30 nM bortezomib (B), and ChIP assays were performed using antibodies for A) total histone 3 (H3), B) total histone 4 (H4), and C) H3K27me3, D) H3K9me3. IgG controls are shown in Fig. 5. Data are presented as percent input and represent a minimum of three independent experiments. No statistically significant differences were found (p>0.05). (TIF) [file pone.0081110.s002.tif]
