# Supplementary material for: The Proteasome Inhibitor Bortezomib Induces an Inhibitory Chromatin Environment at a Distal Enhancer of the Estrogen Receptor-α Gene
Source: PLoS One. 2013 Dec 5;8(12):e81110. doi: 10.1371/journal.pone.0081110 (PMC3855213; doi:10.1371/journal.pone.0081110)
Supplement: Table S1 — Antibodies used for Western Blots. Primary antibodies to the indicated proteins of interest are listed with the specific clone in parenthesis. The Catalog number given is specific for the Company from which the antibody was purchased. The Concentration indicates the dilution of primary antibody in a solution of 5% milk that was used in the Western blot analysis. (DOCX) [file pone.0081110.s003.docx]

**Table S1: Antibodies used for Western Blots**

Protein (clone) Catalog Company Concentration

AP2ɣ (H-77) sc-8977 Santa Cruz 1:2000

β-actin A5441 Sigma 1:5000

ERα (6F11) VP-E613atalog Vector Labs 1:1000

FOXA1(2F83) 39837 Active Motif 1:3000

GATA-3 (HG3-31) sc-268 Santa Cruz 1:1000

HRP-conjugated secondary antibodies:

Mouse-IgG NA931V GE Healthcare Biosciences Corp 1:5000

Rabbit-IgG NA934V GE Healthcare Biosciences Corp 1:5000
