# Supplementary material for: The Proteasome Inhibitor Bortezomib Induces an Inhibitory Chromatin Environment at a Distal Enhancer of the Estrogen Receptor-α Gene
Source: PLoS One. 2013 Dec 5;8(12):e81110. doi: 10.1371/journal.pone.0081110 (PMC3855213; doi:10.1371/journal.pone.0081110)
Supplement: Table S2 — Primers used for Quantitative Reverse Transcriptase PCR (qRT-PCR) of mRNA. Listed are the accession number, location and sequences of primers used for qRT-PCR. Temp. indicates the optimized annealing temperature used for each primer set. (DOCX) [file pone.0081110.s004.docx]

**Table S2: Primers for qRT-PCR of mRNA**

Gene Sequence 5’to 3’ Ref. Temp.
Assession #  *ESR1* 1518f CCTGATGATTGGTCTCGTCTG 60
NM_000125.1 1702r GGCACACAAACTCCTCTCC

*FOXA1* 318f AGGAACTGTGAAGATGGAAGG 60
NM_004496.2 488r ATGTTGCCGCTCGTAGTC

*GATA3* 1429f ACAAAATGAACGGACAGA 60
NM_002051 1511r GTGGTGGTCTGACAGTTC

*RPLP0* (P0) 657f GACAATGGCAGCATCTACAAC [[45](#_ENREF_45)] 60
M17885 759r GCAGACAGACACTGGCAAC

*TFAP2C* (AP2γ) 1009f TGCTTAAATGCCTCGTTACTG 55
NM_003222.3 1100r CCAATCTTGTCCAACTTCTCC
