# Supplementary material for: The Proteasome Inhibitor Bortezomib Induces an Inhibitory Chromatin Environment at a Distal Enhancer of the Estrogen Receptor-α Gene
Source: PLoS One. 2013 Dec 5;8(12):e81110. doi: 10.1371/journal.pone.0081110 (PMC3855213; doi:10.1371/journal.pone.0081110)
Supplement: Table S3 — Primers used for ChIP and DNAse sensitivity assays. Primers used in quantitative PCR analyses of ChIP and DNAse sensitivity assays for the ESR1 gene are shown. The name of the primer indicates its location based on base pair distance 5′ of the TSS. Temp. indicates the optimized annealing temperature for each primer pair. (DOCX) [file pone.0081110.s005.docx]

**Table S3: Primers used for ChIP & DNase sensitivity assays**

Gene Name* Sequence (5’ to 3’) Ref. Temp.
*ESR1* ENH1f -150 kb GAAATCCTTTTCCCCTCTGG [[46](#_ENREF_46)] 60
 ENH1r -150 kb TGGTGCATAAGTGGGAATCA

*ESR1* -811/-940 AGCTGGACCAGACCGACAATG [[39](#_ENREF_39)] 60
 -677/-806 GCCTTCCACAGGTTGGTTATGC

*ESR1* 60/-69 TCCTCCAGCACCTTTGTAATG [[39](#_ENREF_39)] 60
 167/39 AAGTGCAGCTCCCAGGAC

*ESR1* 5307/5179 CAGCCAAAGCGAAAGTGTAAGG [[39](#_ENREF_39)] 60 5462/5334 AGAGACGAAGTAGCGAACAGC

* Location relative to the TSS
