# Supplementary material for: The Proteasome Inhibitor Bortezomib Induces an Inhibitory Chromatin Environment at a Distal Enhancer of the Estrogen Receptor-α Gene
Source: PLoS One. 2013 Dec 5;8(12):e81110. doi: 10.1371/journal.pone.0081110 (PMC3855213; doi:10.1371/journal.pone.0081110)
Supplement: Table S4 — Antibody conditions for ChIP. Shown is a list of antibodies with corresponding catalog number and commercial vendor. Also included is the amount of antibody used for the indicated volume of lysate out of a total of 300 µl of total cell lysate. (DOCX) [file pone.0081110.s006.docx]

**Table S4: Antibody Conditions for ChIP**

Antibody Catalog # Company Amount Lysate^

AcH3 06-599 Millipore 4µL 35µL

AcH4 06-866 Milipore 8µL 35µL

AP2ɣ sc-8977 Santa Cruz 6µg 75 µL

ERα HC-20 sc-543 Santa Cruz 2µg 65µL

FOXA1 sc-22841 Santa Cruz 4µg 65 µL

GATA-3 sc-268x Santa Cruz 2µg 65 µL

H3 07-690 Millipore 2µL 20 µL

H3K9me3 17-625 Millipore 4µL 20 µL

H3K27me3 17-622 Millipore 4µL 30 µL

H4 05-858 Millipore 2µL 20 µL

H4K20me3 07-463 Millipore 10µL 30 µL

IgG (mouse) sc-2025 Santa Cruz variable * variable *

IgG (rabbit) sc-2027 Santa Cruz variable* variable *

p300 sc-584 Santa Cruz 4 µg 65 µL

RNA PolII MMS126R Covance 7µL 65 µL

^ Out of 300 µL total lysate

* Dependent on specific antibody conditions used for comparison
